# seRNA *PAM* controls skeletal muscle satellite cell proliferation and aging through *trans* regulation of *Timp2* expression synergistically with Ddx5

**DOI:** 10.1111/acel.13673

**Published:** 2022-07-18

**Authors:** Karl Kam Hei So, Yile Huang, Suyang Zhang, Yulong Qiao, Liangqiang He, Yuying Li, Xiaona Chen, Mai Har Sham, Hao Sun, Huating Wang

**Affiliations:** ^1^ Department of Chemical Pathology, Li Ka Shing Institute of Health Sciences The Chinese University of Hong Kong Hong Kong SAR China; ^2^ School of Biomedical Sciences The Chinese University of Hong Kong Hong Kong SAR China; ^3^ Department of Orthopaedics and Traumatology, Li Ka Shing Institute of Health Sciences The Chinese University of Hong Kong Hong Kong SAR China; ^4^ Center for Neuromusculoskeletal Restorative Medicine Hong Kong Science Park Hong Kong SAR China

**Keywords:** Ddx5, muscle aging, muscle satellite cell, PAM, seRNA

## Abstract

Muscle satellite cells (SCs) are responsible for muscle homeostasis and regeneration and lncRNAs play important roles in regulating SC activities. Here, in this study, we identify *PAM* (Pax7 Associated Muscle lncRNA) that is induced in activated/proliferating SCs upon injury to promote SC proliferation as myoblast cells. *PAM* is generated from a myoblast‐specific super‐enhancer (SE); as a seRNA it binds with a number of target genomic loci predominantly in *trans*. Further studies demonstrate that it interacts with Ddx5 to tether *PAM* SE to its inter‐chromosomal targets *Timp2 and Vim* to activate the gene expression. Lastly, we show that *PAM* expression is increased in aging SCs, which leads to enhanced inter‐chromosomal interaction and target genes upregulation. Altogether, our findings identify *PAM* as a previously unknown lncRNA that regulates both SC proliferation and aging through its *trans* gene regulatory activity.

## Graphical abstract

Muscle satellite cells (SCs) are indispensable for muscle regeneration and lncRNAs play important roles in regulating SC activities. Here, we characterize the function of lncRNA *PAM* in SC. We show that *PAM* promotes SCs proliferation by binding with Ddx5 to facilitate the chromatin interaction between *PAM* residing super enhancer (SE) and target loci, *Timp2* and *Vim*. In aging mice, the activity of *PAM* SE is elevated to enhance the transcription of *Timp2*, which potentially modulates extracellular matrix (ECM) components in aging muscle.

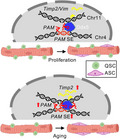

**Abbreviations**
3C: chromatin conformation capture
4C‐seq: chromatin conformation capture sequencing
ASC: activated satellite cell
BET: bromodomain and extraterminal protein family
ChIP‐seq: chromatin immunoprecipitation sequencing
ChIRP‐seq: chromatin isolation by RNA purification sequencing
DNA: deoxyribonucleic acid
eRNA: enhancer RNA
FISC: freshly isolated satellite cell
GO: gene ontology
JQ1: (S)‐Tert‐butyl2‐(4‐(4‐chlorophenyl)‐2,3,9‐trimethyl‐6H‐thieno[3,2‐f][1,2,4]triazolo[4,3‐a][1,4]diazepin‐6‐yl)acetate
lncRNA: long non‐coding RNA
mRNA: messenger RNA
PAM: Pax7 associated muscle lncRNA
QSC: quiescent satellite cell
RNA: ribonucleic acid
rRNA: ribosomal RNA
RT‐qPCR: real time quantitative polymerase chain reaction
SC: satellite cell
SE: super‐enhancer
seRNA: super‐enhancer RNA
siRNA: small interfering RNA

## 1 INTRODUCTION

Skeletal muscle tissue homeostasis and regeneration relies on muscle stem cells, also known as muscle satellite cells (SCs). These cells reside in a niche between the muscle fiber sarcolemma and the basal lamina surrounding the myofiber and are uniquely marked by transcription factor paired box 7 (Pax7). SCs normally lie in a quiescent state, upon activation by injury or disease, the cells quickly activate and express the master myogenic regulator, MyoD, then re‐enter cell cycle and proliferate as myoblasts, subsequently differentiate and fuse to form myotubes (Wang et al., 2014). A subset of SCs undergo self‐renewal and return to quiescence, thus restoring the stem cell pool. Deregulated SC activity contributes to the development of many muscle‐associated diseases. For example, sarcopenia, a highly prevalent elderly disorder condition characterized by declined muscle mass and deficient muscle strength and function, is linked to a progressive reduction in the regenerative capacity of the SCs. It is thus imperative to understand the way SCs contribute to muscle regeneration, and their potential to cell‐based therapies. At cellular level, every phase of SC activity is tightly orchestrated by many molecules and signaling pathways both intrinsically from the cell and extrinsically from the niche; the elucidation of factors and molecular regulatory mechanisms governing SC function thus is of extreme importance, being the first step toward successful use of these cells in therapeutic strategies for muscle diseases.

It has become increasingly clear that long non‐coding RNAs (lncRNAs) are important players regulating SC regenerative activities (Li et al., 2018). For example, *SAM* promotes myoblast proliferation through stabilizing Sugt1 to facilitate kinetochore assembly (Li et al., 2020); *Linc‐YY1* promotes myogenic differentiation and muscle regeneration through interaction with YY1 (Zhou et al., 2015). In another example, we found that in myoblast cells master transcription factor MyoD induces the expression of lncRNAs from super enhancers (SEs), so‐called seRNAs, which in turn regulate target gene expression in *cis* through interacting with hnRNPL (Zhao et al., 2019). In fact, the functional synergism between enhancer‐generated eRNAs and their associated enhancer activity in regulating target promoter expression is well established. There are myriad of mechanisms that eRNAs or lncRNAs cooperate with protein, DNA or RNA partners to regulate transcription of target genes either in *cis* and in *trans*. For example, it is known that specific eRNA can interact with CBP or BRD4 within topologically associating domain (TAD) in a localized manner (Rahnamoun et al., 2018). Similarly, eRNA *Sphk1* evicts CTCF, that insulates between enhancer and promoter, thus activating proto‐oncogene *SPHK1* expression in *cis* (Blank‐Giwojna et al., 2019). Some eRNAs or lncRNAs, on the contrary, play a dual molecular function both in *cis* and in *trans*, for example, *lincRNA‐p21* acts in *trans* by recruiting heterogeneous nuclear ribonucleoprotein K (hnRNPK) to the target promoter (Huarte et al., 2010). Interestingly, in a separate study, *lincRNA‐p21* can also transcriptionally activate *Cdkn1a* in *cis* (Dimitrova et al., 2014). Another well‐demonstrated example of *trans* acting eRNA is *FIRRE*, that interacts with hnRNPU via a conserved Repeating RNA Domain (RRD) to mediate nuclear localization during hematopoiesis (Hacisuleyman et al., 2014; Lewandowski et al., 2019). Also, distal regulatory region of *MyoD* transcribed ^*DRR*^ *eRNA* that interacts with cohesin and transcriptionally activates *Myogenin* in *trans* (Tsai et al., 2018). Altogether, these findings demonstrate the diversified modes of action of eRNAs or lncRNAs in regulating target genes, which needs to be more exhaustively investigated.

Here, in this study, we identify *PAM*, an seRNA that regulates SC proliferation. Expression of *PAM* is evidently upregulated during SC activation/proliferation; consistently, knockdown of *PAM in vitro* hinders SC proliferation. High throughput identification of *PAM* interactome reveals that it regulates tissue inhibitor of metalloproteinases 2 (*Timp2*) locus in *trans* through binding and recruiting Ddx5 protein; loss of *PAM* or Ddx5 results in reduction of chromatin interaction between *PAM* SE and *Timp2* loci. Furthermore, *PAM* SE activity and chromatin connectivity with *Timp2* is elevated in aging SCs; *in vivo* inhibition of the SE activity by JQ1 reduces *Timp2* expression. Altogether, our findings have identified *PAM* as a seRNA regulator of SC proliferation through its *trans* regulation of *Timp2* synergistically with Ddx5.

## 2 RESULTS

### 2.1 lncRNAs profiling identifies *PAM* as a seRNA promoting SC proliferation

**FIGURE 1:**
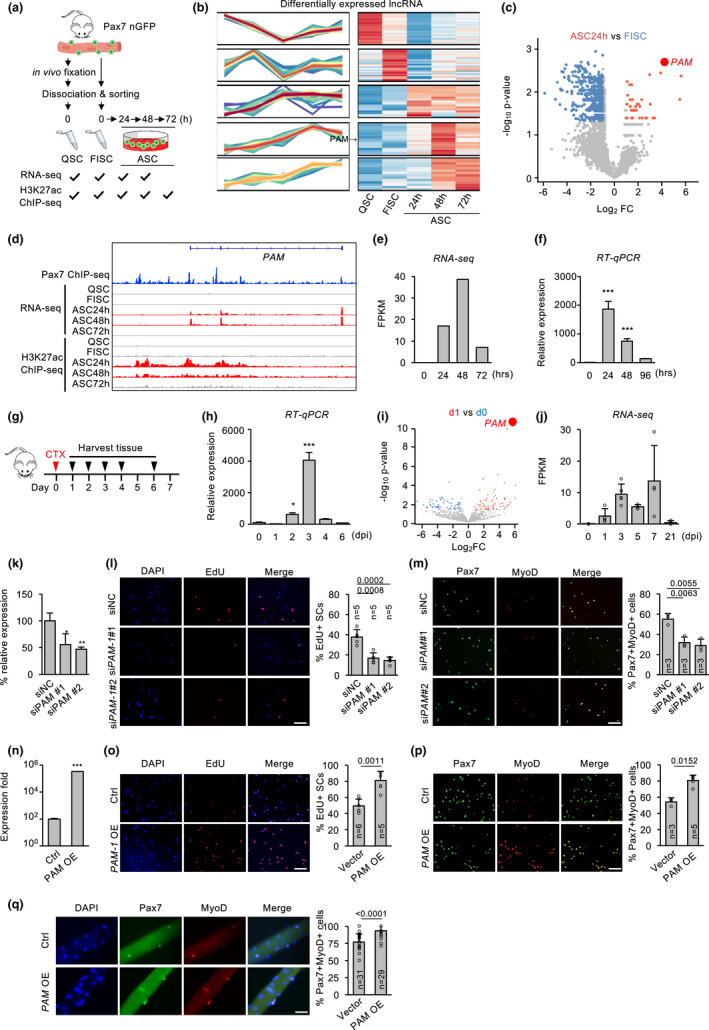
lncRNAs profiling identifies *PAM* as a seRNA promoting SC proliferation. (a) Transcriptomic and epigenomic discovery of lncRNAs in quiescence SCs (QSCs), freshly isolated SCs (FISCs), or activated SCs (ASCs) *in vitro* cultured for 24, 48, and 72 hours (h), respectively. All SCs were isolated from muscles of *Tg:Pax7‐nGFP* mice. (b) Heatmap showing time‐series analysis of differentially expressed lncRNAs during SC lineage progression. *PAM* was highly expressed in ASCs. (c) Volcano plots showing differentially expressed lncRNAs in *in vitro* activated ASC24h vs FISC. *PAM* is indicated in red dot. (d) Genome browser tracks showing temporal expression of *PAM* by RNA‐seq; histone mark H3K27ac and Pax7 ChIP‐seq tracks are also shown. (e) Bar chart showing *PAM* expression dynamics (FPKM) from RNA‐seq data. (f) FISCs were cultured for the designated time and *PAM* expression was detected. (g) Schematic illustration of in acute muscle injury‐induced regeneration. CTX was injected into TA muscles of *Tg:Pax7‐nGFP* mice and FISCs were collected at the designated days post‐injury (dpi). (h) RT‐qPCR detection of *PAM* in the above FISCs. (i) Publicly obtained RNA‐seq data from SCs collected at 0, 1, 3, 5, 7, or 21 dpi were analyzed. Volcano plots showing differentially expressed lncRNAs in SCs from Day 1 vs. Day 0 post‐injury. (j) Bar chart showing *PAM* expression in FPKM from the above data. (k) ASCs were transfected with either control (siNC) or *PAM* siRNA (siPAM#1 or siPAM#2) and *PAM* knockdown was confirmed by RT‐qPCR 48 h post‐transfection. (l) EdU incorporation assay was performed in the above cells and the percentage of EdU+ cells were quantified. (m) Pax7+/MyoD+ immunostaining was performed in the above cells and double positively stained cells were quantified. (n) ASCs were transfected with a *PAM* expression plasmid and *PAM* overexpression was confirmed 48 h post‐transfection (o,p) EdU incorporation assay (o) or Pax7+/MyoD+ immunostaining (p) were performed in the above cells. The percentage of EdU+ or Pax7+/MyoD+ cells were quantified. (q) Overexpression of *PAM* in freshly isolated myofibers increased the percentage of Pax7+/MyoD+ cells 48 h post‐transfection. The percentage of EdU+ and Pax7+/MyoD+ cells were quantified. The total number of biologically independent samples are indicated in (l,m, o,q). (data represent the mean ± SD. *p*‐value was calculated by two‐tailed unpaired *t* test [*p < 0.05, **p < 0.01, ***p < 0.001]. Scale bars: 100 μm)

To gain global insights into the catalog of lncRNAs in the course of SC lineage progression, we analyzed our recently generated RNA‐seq datasets (Zhao et al., 2021) from freshly isolated satellite cells (FISC) which are believed to be early activating by the isolation process, FISCs cultured for 24, 48, or 72 hours (h) to become activated (ASC24h), proliferating (ASC48h) and differentiating (ASC72h) (Figure 1a). We also included the published RNA‐seq dataset from quiescent satellite cells (QSCs) which were in situ fixed in ice‐cold 0.5% paraformaldehyde before cell dissociation to preserve their quiescence (Machado et al., 2017). The analysis revealed transcriptomic signatures that were consistent with the SC lineage progression timing (Zhao et al., 2021). A principal component analysis (PCA) also showed that QSC and FISC had similar transcriptomic signatures while ASC time points were more similar to each other (Figure S1a). To identify lncRNAs differentially expressed during the lineage progression, we found a total of 143 lncRNAs were dynamically expressed in QSC, FISC, and ASCs (Figure 1b). To further identify lncRNA candidates that may play a role in promoting SC activation/proliferation, we found a total of 40 lncRNAs were upregulated in ASC24h vs FISC (Figure 1c and Table S1) and 48 in ASC48h vs FISC (Figure S1b and Table S1). Among these differentially expressed lncRNAs, 21 were upregulated in both ASC24h and ASC48h (Figure S1c and Table S1). *Gm12603* was among the highest induced ones; it was barely detectable in QSC and FISC but highly induced in activating (17.9‐fold) and proliferating myoblasts (39.6‐fold) (Figure 1d,e). The *Gm12603* expression calculated from the RNA‐seq data was also confirmed by RT‐qPCR using *in vitro* cultured SCs (Figure 1f). Indeed, it was induced when SCs were activated after culturing for 24 h (1856‐fold), the expression remained high at 48 h (746‐fold) but dropped at 96 h when cells differentiated (Figure 1f). The above finding was also validated *in vivo* by examining *Gm12603* expression in acute injury induced regenerating muscles (Chen et al., 2021). Cardiotoxin (CTX) was injected into tibialis anterior (TA) muscle in young adult (2–4 months old) at Day 0, and SCs were sorted out at 1, 2, 3, 4, and 6 days post‐injury (dpi) for RT‐qPCR (Figure 1g). As expected *Gm12603* expression was induced at 2 dpi and peaked at 3 dpi (Figure 1h) concomitant with the SC activation/proliferation phase *in vivo* (Shcherbina et al., 2020). The *in vivo* expression dynamics of *Gm12603* was also confirmed by analyzing publicly available RNA‐seq data generated from SCs collected in regenerating muscles (Figure 1i,j, S1d). Altogether, the above data demonstrate a possible role of lncRNA *Gm12603* in promoting SC activation/proliferation. Interestingly, we also found it was bound by Pax7 at its promoter region via analyzing the publicly available Pax7 ChIP‐seq data in myoblasts (Soleimani et al., 2012), further confirming its functional relevancy in SCs. We thus re‐named it Pax7 associated muscle (*PAM*) lncRNA.

*PAM* is a lncRNA located on chromosome 4: in the intervening region of Interferon alpha (*Ifna*) family and Cyclin‐dependent kinase 2 inhibitor (*Cdkn2a* and *Cdkn2b*) protein‐coding genes. It is also known as *Wincr1*, a Wnt activated lncRNA in mouse dermal fibroblast affecting extracellular matrix composition via collagen accumulation in dermal fibrosis (Mullin et al., 2017). To dissect its function in SCs, we next cloned its sequence from C2C12 myoblast cell line by Rapid Amplification of cDNA ends (RACE); it was 718 bp long with three exons. More interestingly, we found that *PAM* is generated from a SE region defined using our published H3K27ac ChIP‐seq datasets (Figure 1d) (He et al., 2021). Concomitant with the expression pattern of *PAM*, high level of H3K27ac ChIP‐seq signals were observed in ASC24h and ASC 48 h but not in FISC (Figure 1d).

The above findings suggested that *PAM* may function as a seRNA to promote SC activation/proliferation. To test the potential function of *PAM* in myoblast proliferation, we knocked down *PAM* in FISCs from young adult (2–4 months old) with two separate siRNA oligos and performed EdU incorporation after 2 days; a 21 and 23% decrease of EdU+ cells were observed upon *PAM* knockdown (Figure 1k,l); similarly, by staining for Pax7 and MyoD 48 h after transfection, Pax7+/MyoD+ cells were also significantly reduced by 22% and 25% (Figure 1m). Gain‐of‐function assay was also performed by overexpressing a *PAM* plasmid in FISCs and increased proliferation was observed by both EdU+ (32% increase) and Pax7+/MyoD+ (26% increase) cells (Figure 1n,p). Altogether, the above results from cultured cells suggested *PAM* indeed promotes SC proliferation. Similar conclusion was also obtained using in SCs associated with isolated and cultured single muscle fibers; overexpressing *PAM* led to a 26.66% increase of Pax7+/MyoD+ cells (Figure 1q).

### 2.2 *PAM* interacts with inter‐chromosomal loci to modulate target expression

**FIGURE 2:**
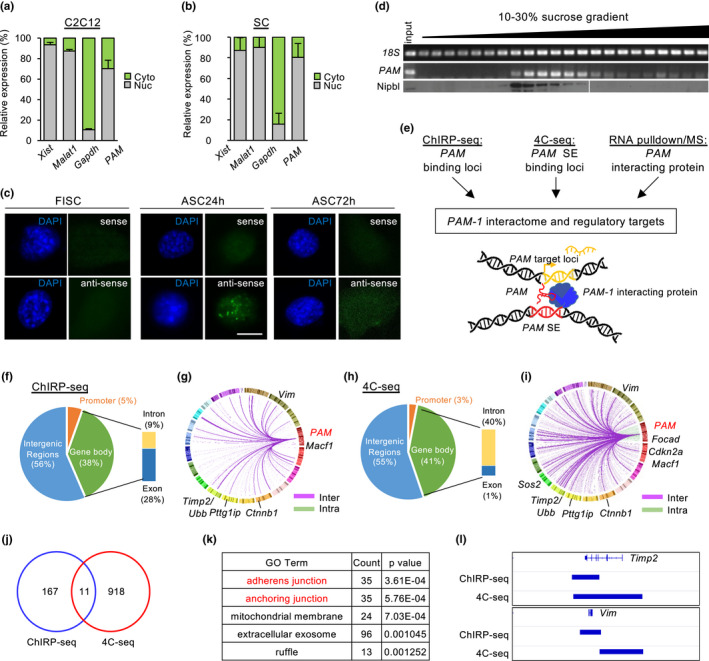
*PAM* is a nuclear‐retained lncRNA, forming *cis* and *trans* chromosomal interactions (a,b). Cellular fractionation of (a) C2C12 cell line and (b) ASC showed *PAM* was enriched in nucleus not cytosolic fraction. *Xist* and *Malat1* were positive control for nuclear fraction, *Gapdh* was positive control for cytosolic fraction. (c) Fluorescence in situ hybridization (FISH) in FISC, ASC24h and ASC72h using *PAM* antisense (AS) probe showed nuclear localization of *PAM*, sense probe (S) was used as negative control. Scale bar: 10 μm (d) Cellular fractionation using sucrose gradient ultracentrifugation showed *PAM* was co‐localized in fractions containing nuclear protein Nipbl. (e) Experimental workflow to discover *PAM* interactome and regulatory targets. For details and controls, see experimental procedures. (f) Pie chart showing distribution of *PAM* seRNA interacting chromatin across the genome in ChIRP‐seq. (g) Circos plot showing genes associated to *PAM* seRNA interacting chromatin, each line in the plot represents an interaction, line colors represent inter‐chromosomal (purple) or intra‐chromosomal (green) interactions. Chromosome numbers were colored and arranged in clockwise direction. Top‐ranked genes were named in the figure. (h) Pie chart showing distribution of *PAM* SE interacting chromatin across the genome in 4C‐seq. (i) Circos plot showing genes associated to *PAM* SE interacting chromatin, each line in the plot represents an interaction, line colors represent inter‐chromosomal (purple) or intra‐chromosomal (green) interactions. Chromosome numbers were colored and arranged in clockwise direction. Top‐ranked genes were named in the figure. (j) Venn diagram showing overlapping loci between ChIRP‐seq of *PAM* seRNA and 4C‐seq of *PAM* SE. (k) Table showing top‐ranked gene ontology (GO) terms of genes associated with overlapping loci from ChIRP‐seq of *PAM* seRNA and 4C‐seq of *PAM* SE. (l) Genome browser tracks showing *Timp2* and *Vim* as examples of *PAM* inter‐chromosomal targets. ChIRP‐seq tracks indicate regions of broad peaks enriched with *PAM* seRNA binding. 4C‐seq tracks indicate fragments cut by two restriction enzymes and showing significant chromatin interaction with *PAM* SE

To further elucidate the regulatory mechanism of *PAM* in SC proliferation, we sought to identify the subcellular localization pattern of *PAM* as lncRNA function is largely determined by its cellular localization (Yao et al., 2019) and seRNAs are known to be localized in both nucleus and cytoplasm of muscle cells (Zhao et al., 2019). Cellular fractionation was performed using ASC (Figure 2a) and *PAM* was found to localize largely in nuclear fraction (70.21%); as controls, lncRNAs *Xist* and *Malat1* were predominately nuclear‐localized (93.61% and 87.37%, respectively) while *Gapdh* mRNAs were enriched in the cytoplasm (89.66%). Similar phenomena were also found when performing cellular fractionation in C2C12 myoblast (Figure 2b). The above finding was further validated by RNA Fluorescence in situ hybridization (FISH) using *PAM* antisense probe, which also revealed *PAM* transcript was predominantly enriched in the nucleus of ASC24h but barely detectable in FISC or ASC72h (Figure 2c). Lastly, Subcellular localization of *PAM* was also confirmed using sucrose gradient centrifugation on C2C12 myoblast lysate which separated protein complex based on their size. Using cohesin loading factor NIPBL as positive control for nuclear fraction, we also found *PAM* mainly enriched in the nucleus of myoblasts (Figure 2d). Taken together, our results show *PAM* is a nuclear enriched seRNA.

To elucidate the functional mechanism of *PAM* as a nuclear seRNA, we reasoned it could function to regulate target gene transcription as an integral component of *PAM* residing SE. Considering SE and seRNA are known to work synergistically by tethering the active transcriptional machinery to target loci, we decided to identify *PAM* seRNA and *PAM* SE interacting loci on the genome, respectively (Figure 2e). We first performed *PAM* Chromatin Isolation by RNA Purification (ChIRP‐seq) in ASCs to identify its binding target loci. As a result, we found that *PAM* seRNA interacted with 178 DNA regions across the genome which were mainly distributed in intergenic (56%) and gene body regions (36%) (Figure 2f); and these regions were associated with 1199 genes that were enriched for GO terms such as “fiber association,” “actin filament organization,” and “regulation of cell shape” et al. (Figure S2a and Table S2). Strikingly, *PAM* was dominantly associated with inter‐ but not intra‐chromosomal regions (Figure 2g). Only 5 out of the 178 regions were found on chromosome 4 and not adjacent to *PAM* gene locus (Table S2). The above findings suggested *PAM* seRNA may predominantly target gene loci in *trans*. Next, we set out to identify *PAM* SE target genes by performing circular chromosome conformation capture (4C‐seq) using *PAM* SE region as a bait to query genome‐wide chromatin interactions. Among 929 interacting targets, 74% (687) were in *trans* while only 26% (242) were in *cis* on chromosome 4 (Figure 2h,i and Table S3).

To further define target loci co‐regulated by *PAM* and its SE region, we next integrated the targets from the above ChIRP‐seq and 4C‐seq and identified 11 common loci in both datasets (Figure 2j); one of the loci was located 33 Mb downstream of *PAM* on chromosome 4, while others were all located in other chromosomes (Figure S2b), suggesting *PAM* and *PAM SE* may regulate genes in *trans* together. These 11 loci were associated with 152 genes, which were enriched for GO terms such as “adherens junction,” “anchoring junction” (Figure 2k). *Timp2* and *Vimentin* (*Vim*) genes were among the top‐ranked (Figure 2l). Timp2 plays a dual role in mediating extracellular matrix by mediating matrix metalloproteinase (MMP) activation and inhibition via interaction with MMP‐14 and MMP‐2 (Young et al., 2004). Overexpression of *Timp2* in C2C12 myoblasts was known to delay myogenic differentiation and arrest C2C12 in proliferating state (Lluri & Jaworski, 2005). Vim is an intermediate filament protein to modulate cell shape and motility in myoblast, which is also considered as a reliable marker for regenerating muscle tissue (Bornemann & Schmalbruch, 1992).

### 2.3 *PAM* seRNA and *PAM* residing SE synergistically activate *Timp2* and *Vim* transcription in myoblast

**FIGURE 3:**
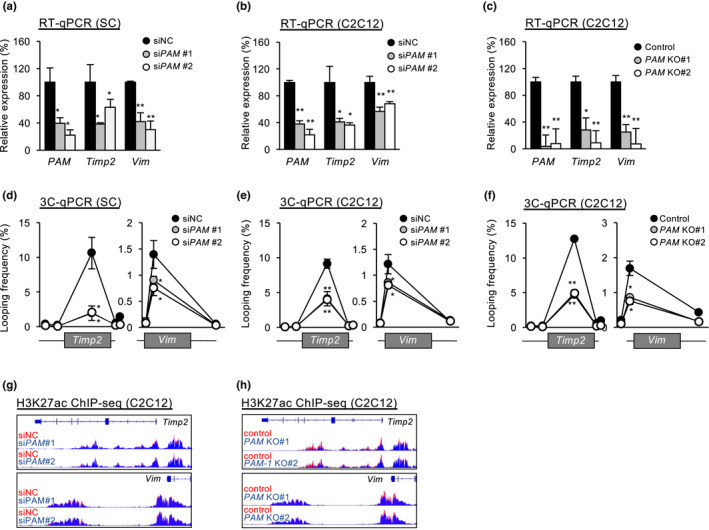
*PAM* regulates extracellular matrix‐associated genes *Timp2* and *Vim*. (a,b) ASCs (a) or C2C12 (b) were transfected with either control (siNC) or *PAM* siRNA (siPAM#1 or siPAM#2) oligos, the expression levels of *PAM*, *Timp2* and *Vim* were detected 48 hours post‐transfection. (c) RT‐qPCR detection of *PAM*, *Timp2* and *Vim* from C2C12 with or without *PAM* knockout. (d) ASCs (d) or C2C12 (e) were transfected with either control (siNC) or *PAM* siRNA (siPAM#1 or siPAM#2) oligos and chromatin conformation capture assay (3C‐qPCR) was performed to detect the chromatin interaction between *PAM* SE with *Timp2* or *Vim* locus (f) the above 3C‐qPCR was performed in C2C12 cells with *PAM* knockout. (g,h) Genome browser tracks showing both knockdown (g) and knockout (h) of *PAM* led to reduction in H3K27ac signal intensity on *Timp2* and *Vim* loci. Data information: Data represent the mean ± SD. *p*‐value was calculated by two‐tailed unpaired *t*‐test (*p < 0.05, **p < 0.01)

The above findings raised an intriguing possibility that *PAM* seRNA and *PAM* SE interact with the inter‐chromosomal target loci *Timp2* and *Vim* to activate their transcription (Figure 2e). Consistent with the notion, we found that *PAM*, *Timp2*, and *Vim* expressions were upregulated during SC activation/proliferation (Figure S2c). In addition, knocking down *PAM* seRNA using siRNA oligo decreased the expression levels of *Timp2* and *Vim* in both ASCs and C2C12 myoblasts (Figure 3a,b); removing the *PAM* SE region using CRISPR/cas9 in C2C12 (Figure S2d) yielded similar molecular phenotype (Figure 3c). To test the possibility that *PAM* functions to tether *PAM* SE to the target loci, we performed 3C qPCR assay in ASCs and C2C12 myoblasts; in line with the 4C‐seq result (Figure 2h), *PAM* locus indeed displayed evident interaction with *Timp2* and *Vim* promoters (Figure 3d,e); Furthermore, *PAM* siRNA oligo mediated knockdown significantly reduced the interaction with *Timp2* and *Vim* (Figure 3d,e and S2e). Consistently, *PAM* SE knockout also yielded reduction in chromatin interactions with *Timp2* and *Vim* (Figure 3f and S2e). Lastly, knockdown of *PAM* of *PAM* SE also led to subtle reduction in H3K27ac signals on *Timp2* and *Vim* in C2C12 myoblasts (Figure 3g and S2f), which was more evidently shown in *PAM* knockout (Figure 3h). Altogether, our results demonstrate that *PAM* seRNA and *PAM* SE can indeed interact with inter‐chromosomal target loci *Timp2* and *Vim* to modulate their expression.

### 2.4 *PAM* regulates inter‐chromosomal targets via associating with Ddx5

**FIGURE 4:**
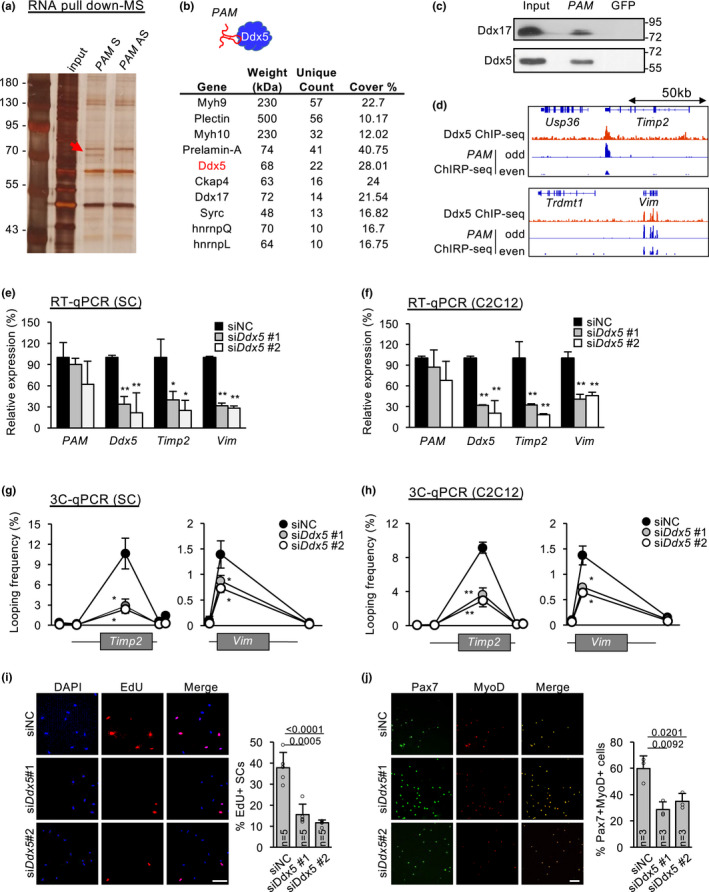
*PAM* regulates inter‐chromosomal targets via association with Ddx5. (a) RNA pulldown using *PAM* sense (S) or antisense (AS) experiment followed by SDS‐PAGE. AS was used as negative control. Red arrow indicates enrichment of a protein band at 70 kDa specifically found in pulldown using *PAM* sense probe. (b) Mass spectrometry (MS) result of the above band showing a list of potential protein binding partners of *PAM*. (c) RNA pulldown followed by Western blotting of the above identified two candidate protein partners (Ddx5 and Ddx17) of *PAM‐* transcript. (d) Genome browser tracks showing Ddx5 ChIP‐seq and *PAM* ChIRP‐seq on *Timp2* or *Vim* locus. (e,f) ASCs (e) or C2C12 (f) were transfected with either control (siNC) or *Ddx5* siRNA (siDdx5#1 or siDdx5#2) oligos, *PAM*, *Ddx5*, *Timp2* and *Vim* expression levels were detected by RT‐qPCR 48 h post‐transfection. (g,h) ASCs or C2C12 were transfected with either control (siNC) or *Ddx5* siRNA (siDdx5#1 or siDdx5#2) oligos, 3C qPCR was performed to detect chromatin interaction between *Timp2* or *Vim* promoter with *PAM* locus. (i,j) Knockdown of *Ddx5* expression *in vitro* in cultured ASCs for 48 hours led to reduction in EdU+ (i) or Pax7+/MyoD+ (j) SCs. The percentage of EdU+ and Pax7 + Myod+ cells were quantified. The total number of biologically independent samples are indicated in (i,j). Data information: Data represent the mean ± SD. *p*‐value was calculated by two‐tailed unpaired *t* test (*p < 0.05, **p < 0.01). Scale bar: 100 μm

To further explore molecular mechanism on how *PAM* regulates its inter‐chromosomal targets *Timp2* and *Vim*, we performed RNA pulldown followed by mass spectrometry (MS) to identify its protein interactome (Figure 4a). Biotinylated sense probe of *PAM* was used in the RNA pulldown *in vitro*, while biotinylated antisense probe was used as negative control for non‐specific protein binding. Among 134 potential protein partners retrieved by the sense probe with at least 5 unique peptide counts (Figure 4b), two known RNA binding proteins (RBPs), DEAD‐Box Helicase 5 (Ddx5) and DEAD‐Box Helicase 17 (Ddx17) were highly ranked showing 22 and 14 unique peptide counts, respectively (Figure 4b). These helicase proteins are commonly found to bind together in multiple cell types to carry out a myriad of molecular functions such as transcription regulation, rRNA processing, mRNA decay, and splicing (Giraud et al., 2018). To validate the above result, we performed RNA pulldown followed by Western blot. *PAM* seRNA but not GFP control transcripts retrieved an evident amount of Ddx5 and Ddx17 from C2C12 myoblasts, with Ddx5 showing a stronger binding with *PAM* seRNA (Figure 4c). To test if Ddx5 regulates target expression in cooperation with *PAM* seRNA, we first performed Ddx5 ChIP‐seq which revealed an evident binding of Ddx5 on *Timp2* and *Vim* loci (Figure 4d), suggesting *PAM* and Ddx5 are both tethered to the target loci. Consistent with their possible functional synergism, knockdown of *Ddx5* in ASC or C2C12 led to down‐regulation of *Timp2* and *Vim* (Figure 4e,f). Furthermore, the above knockdown also reduced the interaction between *PAM* SE and the promoters of *Timp2* and *Vim* (Figure 4g,h), suggesting Ddx5 indeed promotes the inter‐chromosomal interaction together with *PAM*. Lastly, knockdown of *Ddx5* reduced ASC proliferation as revealed by reduction in both EdU+ (Figure 4i) and Pax7+/MyoD+ cells (Figure 4j). Taken together, our findings demonstrate *PAM* seRNA and Ddx5 function synergistically in orchestrating the inter‐chromosomal interactions between the *PAM* SE with the two target loci, *Timp2* and *Vim*, consequently promoting transcriptional activity.

### 2.5 *PAM* upregulation in aging SCs drives its target gene activation

**FIGURE 5:**
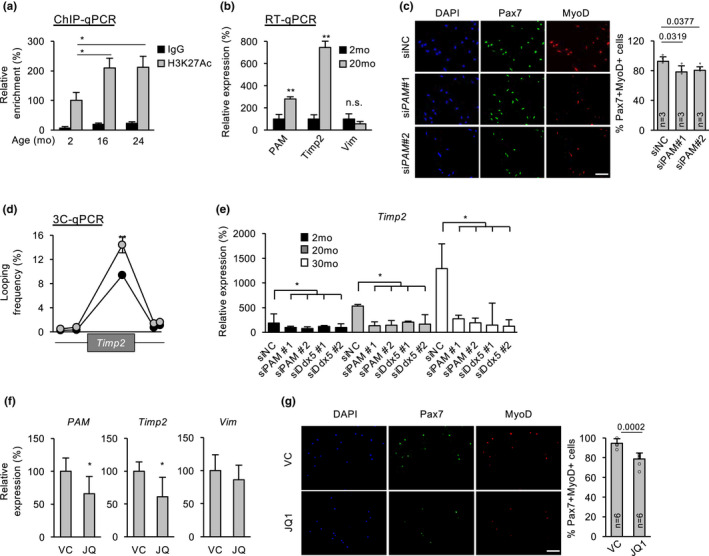
*PAM* increase in aging SCs drives its target gene upregulation. (a) H3K27ac ChIP‐qPCR showing increase in enrichment on *PAM* SE in ASCs isolated from aging (16 and 24 months) vs young (2 months) old mice. (b) RT‐qPCR showed upregulation of *PAM* and target genes *Timp2* but not *Vim* in ASCs from 20 vs 2 month old mice. (c) Knockdown of *PAM* in aging ASC from 20 months old mice reduced the number of Pax7+/MyoD+ proliferating ASC. (d) 3C‐qPCR assay showed increase in interaction between *PAM* locus and *Timp2* promoter in the above aging ASCs. (e) RT‐qPCR showing the expression dynamics of *Timp2* in ASCs from 2, 20 and 30 months old mice. Knockdown of *PAM* or *Ddx5* using siRNA oligo in ASCs from 20 or 30 months old mice showed down‐regulation of *Timp2*. (f,g) *in vivo* treatment of JQ1, a Brd4 inhibitor, in 10 month old mice caused down‐regulated expression of *PAM* and *Timp2* but not *Vim* in FISCs (f), and reduced number of Pax7+/MyoD+ (g) in aging SC. The total number of biologically independent samples are indicated in (c&g). Data information: Data represent the mean ± SD. *p*‐value was calculated by two‐tailed unpaired *t* test (*p < 0.05, **p < 0.01). Scale bar: 100 μm

Lastly, to test the possible involvement of *PAM* in SC aging, we isolated SCs from mice of various ages (2, 16, or 24 months) and cultured for 48 h to obtain proliferating ASC. By performing H3K27Ac ChIP‐qPCR, we found the activity of *PAM* SE was indeed increased by 109% in ASC from 16 month as compared with 2‐month‐old mice, and continued to increase at 24 month (Figure 5a). The expression of *PAM* and *Timp2* also showed an increase (308% and 681%) in 20 vs 2‐month ASC. Interestingly, *Vim* expression remained unchanged (Figure 5b); the lack of response in *Vim* could be due to the weak interaction between *Vim* locus and *PAM* SE (Figure 3d). In addition, we also obtained RNA‐seq data from injured 2‐ or 24‐month‐old muscles (Shcherbina et al., 2020) and found *PAM* was increased endogenously in ASC isolated 1 dpi from 24 vs 2‐month‐old mice (Figure S3a). Knockdown of *PAM* in ASCs from 20‐month‐old muscle also led to reduced proliferation (Figure 5c and S3b). The above data thus suggest that both *PAM* SE activity and *PAM* expression is elevated in aging SCs and may contribute to the upregulated *Timp2* expression. Consistent to the notion, the interaction between *PAM* locus and the promoter of *Timp2* were found to increase by 46.75% in 20 vs 2‐month ASC (Figure 5d). Lastly, to further confirm the synergistic function of *PAM* and Ddx5 in increasing *Timp2* expression in aging SCs, we found that knockdown of *PAM* or *Ddx5* in ASC in 2‐, 20‐ or 30‐month‐old mice led to down‐regulation of *Timp2* (Figure 5e).

Recently, we have reported the use of BET family of bromodomain protein binding inhibitor JQ1 to down‐regulate enhancer activity in aging mouse muscle tissue (Zhou et al., 2019). Expectedly, we found that intraperitoneal injection of JQ1 in 10‐month‐old mice led to a down‐regulation of *PAM* seRNA and *Timp2* expression in FISCs, but not *Vim* (Figure 5f). Consistently, we found ASC proliferation was reduced by JQ1 treatment (16% in Pax7 + MyoD+ cells) (Figure 5g). Altogether, our data reinforce the notion that *PAM* SE activation causes *Timp2* upregulation during SC aging.

## 3 DISCUSSION

**FIGURE 6:**
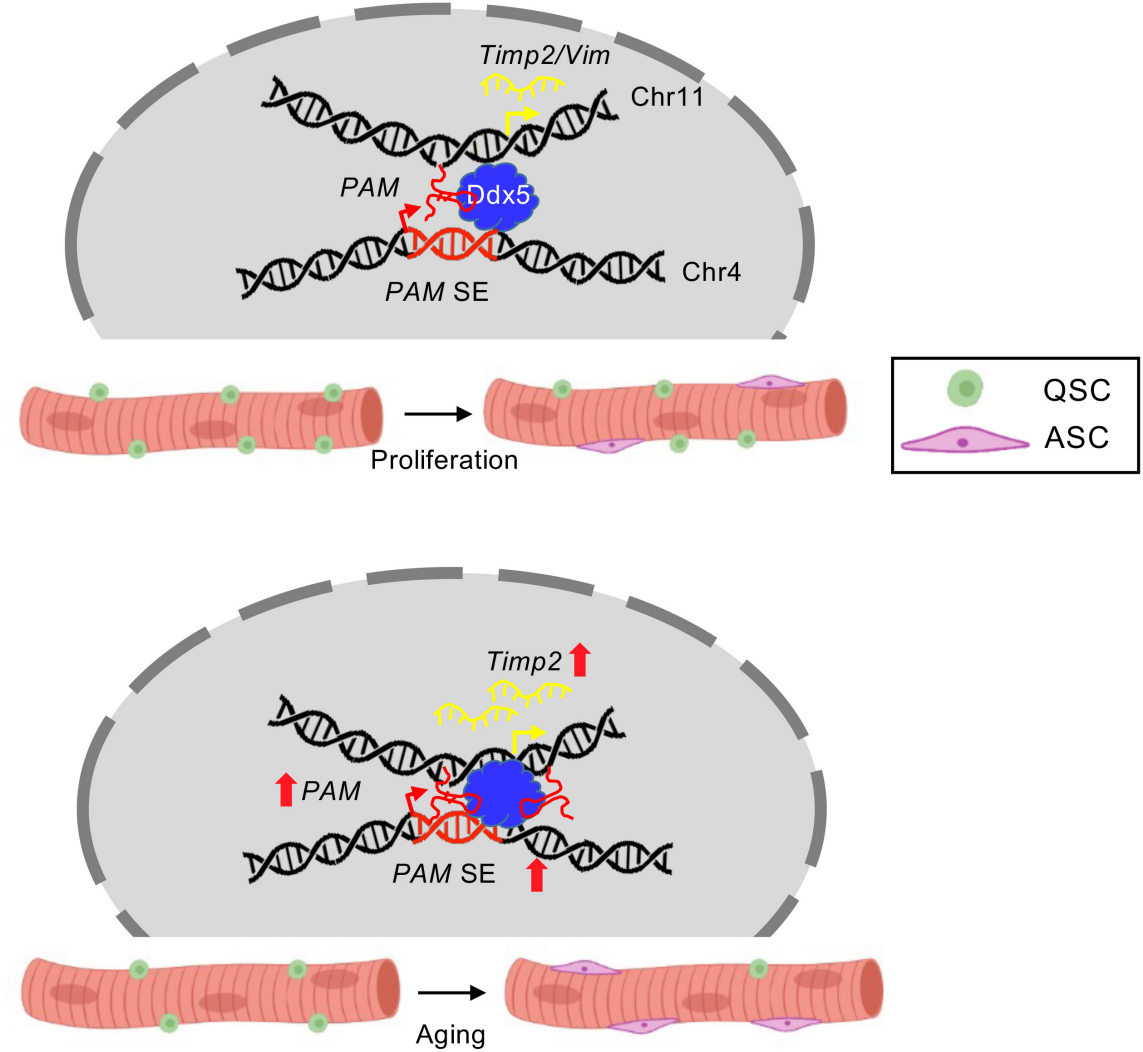
Schematic model showing functional role of seRNA *PAM* in SC proliferation and skeletal muscle aging. *PAM* regulates SCs proliferation by binding with Ddx5 to facilitate the chromatin interaction between *PAM* SE and target loci, *Timp2* and *Vim* during SC proliferation. In aging mice, the activity of *PAM* SE was elevated, thereby enhancing the transcription of *Timp2*, which potentially modulates extracellular matrix components in skeletal muscle

Myogenesis is a complex process that relies on tightly regulated and finely tuned transcriptional regulatory mechanisms. Previous studies have discovered a myriad of lncRNAs that are dynamically regulated during myogenesis (Alessio et al., 2019). Yet, their functional mechanisms in SCs remain largely unexplored. Here, in this study, we identified *PAM*, a seRNA that binds with Ddx5 protein to synergistically tether the SE to its inter‐chromosomal target loci *Timp2* and *Vim*. Furthermore, we showed that deregulation of *PAM* in aging SCs drives the target gene deregulation (Figure 6).

Through transcriptomic profiling, *PAM* was identified as highly induced in activated and proliferating SCs. Loss‐ and gain‐of‐function studies in SCs indeed pinpointed it as a promoting factor for SC proliferation. Its nuclear localization is consistent with its nature of being a seRNA. As part of the SE regulatory machinery, *PAM* and its residing SE together promote the expression of their target loci, *Timp2* and *Vim*, both encoding extracellular matrix proteins. Increased extracellular matrix (ECM) proteins expressions are essential to SC activation (Zhang et al., 2021). The regulation of ECM composition was conventionally believed to be mediated by surrounding fibroblasts, fibro/adipogenic progenitors, myofibers, and basal lamina. In addition to receiving signals from ECM, emerging evidence demonstrates SCs also contribute to ECM compositions through secretion of matrix metalloproteases and urokinase plasminogen activator (Guerin & Holland, 1995). For example, transcriptome profiling of freshly isolated SCs revealed that cell adhesion and ECM genes, such as *Timp* and integrin, were differentially expressed when compared with freshly isolated SCs from dystrophic mdx mice (Pallafacchina et al., 2010). This correlation was further demonstrated by systemic delivery of MMP inhibitor, AM409, which impairs SC activation (Pallafacchina et al., 2010). Therefore, upregulation of ECM gene expression mediates the promoting function of *PAM* during SC proliferation. Furthermore, we showed that *PAM* upregulation contributes to ECM increase in aging SCs. It is known that the regenerative potential of SCs declines during aging, which is in concurrent with its fibrogenic conversion and muscle fibrosis (Yamakawa et al., 2020). Using publicly available single‐cell RNA‐seq analysis, ECM and SC niche are contributed by multiple cell types, while *Timp2* is expressing in SCs, macrophages, mesenchymal stem cells, and endothelial cells (Tabula Muris, 2020) (Figure S3c). Our findings thus provide a potential way to partially restore ECM in aging SCs by down‐regulating *PAM* expression in a cell‐autonomous manner. While our data suggest an elevated *PAM* expression in aging SCs, it remains to be determined whether this is due to faster activation of aged SCs or not. In the future, more efforts will be needed to elucidate the potential roles of lncRNAs in aging SCs. For example, a recent study on integrated transcriptome analysis of aging human skeletal muscle revealed a group of differentially expressed lncRNAs, and overexpression of lncRNA *PRKG1‐AS1* could increase cell viability and reduce apoptosis in human skeletal myoblast (Zheng et al., 2021).

Mechanistically, our data highlight the important role of *PAM* seRNA to regulate inter‐chromosomal targets through tethering its residing SE to the target loci. eRNAs or seRNAs are commonly known to regulate enhancer‐promoter interactions as an integrated component of SE activating machinery (Zhao et al., 2019); it is believed that *cis* regulation of the target loci through intra‐chromosomal interactions is a more prevalent mode compared with *trans* regulation via inter‐chromosomal interactions (Sartorelli & Lauberth, 2020). However, *trans* activating eRNAs do exist to translocate to distal chromosomal regions beyond its neighboring loci. For example, *MyoD* distal enhancer generates ^*DRR*^ *eRNA* that transcriptionally regulates *Myogenin* expression in *trans* via cohesin recruitment (Tsai et al., 2018). Similarly, in human prostate cancer, an adjacent eRNA of kallikrein‐related peptidase 3 (*KLK3*) can regulate target genes expression in *trans* (Hsieh et al., 2014). Our findings from integrating ChIRP‐seq and 4C‐seq demonstrate that *PAM* mainly acts in *trans* to exert its regulatory function in SCs; thus, provide additional evidence to support the *trans* regulatory mechanism by eRNAs.

Another important discovery from our study stems from the identification of a direct physical interaction between *PAM* and Ddx5. Ddx5 and *PAM* synergistically facilitate the SE‐target interaction; knockdown of *Ddx5* impaired the interaction. Although classically known as a RNA helicase controlling mRNA splicing, recent studies demonstrated Ddx5 interacts with a myriad of lncRNAs, and uses the lncRNAs as a scaffold to bring in specific transcriptional machinery or chromatin architectural protein in context‐dependent manner (Giraud et al., 2018). It was also demonstrated that Ddx5 interacts with lncRNA *mrhl* to mediate cell proliferation in mouse spermatogonia cells (Arun et al., 2012). Ddx5 and Ddx17 (p68 and p72) bind with lncRNA *SRA* in regulating skeletal muscle differentiation (Caretti et al., 2006). With foundation laid by these studies, we further demonstrated the functional role of Ddx5 in mediating chromatin interactions via its interaction with *PAM*, underscoring the prevalence of lncRNAs and RNA helicase interaction and broadening the mechanisms through which lncRNAs regulate gene expression.

## 4 EXPERIMENTAL PROCEDURES

### 4.1 Mice

*Tg:Pax7‐nGFP* mouse strain (Sambasivan et al., 2009) was kindly provided by Dr. Shahragim Tajbakhsh. All animal handling procedures and protocols were approved by the Animal Experimentation Ethics Committee (AEEC) at the Chinese University of Hong Kong.

### 4.2 JQ1 treatment

JQ1 treatment was performed as described previously (Zhou et al., 2019). *C57/BL6* mice were caged in groups of five and maintained at controlled temperature (20 ± 1°), humidity (55 ± 10%), and illumination with 12 h light/ 12 h dark cycle. Food and water were provided ad libitum. All procedures involving animal care or treatments were approved by the Animal Ethics Committee (AEEC) at Chinese University of Hong Kong (CUHK) on the protection of animals used for scientific purposes. To investigate the effect of JQ1 on aging skeletal muscle, daily intraperitoneal injection of JQ1 at 50 mg/kg was performed on 10‐month‐old mouse for 14 days (Zhou et al., 2019), with DMSO as control. Then, TA muscle tissues were extracted from mice, and SCs were isolated from mouse skeletal muscle by fluorescence‐activated cell sorting (FACS) using BD FACSAria Fusion cell sorter (BD Biosciences) with cell surface marker Sca1^−^/CD31^−^/CD45^−^/Vcam^+^ (Chen et al., 2019).

### 4.3 Cells

Mouse C2C12 myoblast cell (CRL‐1772) was obtained from American Type Culture Collection (ATCC) and cultured in DMEM medium with 10% fetal bovine serum, 1% penicillin/streptomycin at 37°C in 5% CO_2_. Oligonucleoties of siRNA against mouse *PAM* and scrambled control were obtained from Ribobio Technologies (Guangzhou, China). siRNAs were transfected at 100 nM into C2C12 using Lipofectamine 2000 (Life Technologies). The sequences of oligonucleotides using for siRNA knockdown were listed in Table S4.

### 4.4 Satellite cell isolation and culture

Hindlimb skeletal muscles from *Tg:Pax7‐nGFP* mice were dissected and minced, followed by digestion with Collagenase II (LS004177, Worthington, 1000 units/ml) for 90 min at 37°C in water bath shaker. Digested muscles were then washed in washing medium (Ham's F‐10 medium (N6635, Sigma) containing 10% heat‐inactivated horse serum (Gibco, 26,050,088) with 1% penicillin/ streptomycin, followed by incubating in digestion medium with Collagenase II (100 units/ml) and Dispase (1.1 unit/ml, Gibco, 17,105–041) for additional 30 min. Suspensions were then passed through 20G syringe needle to release myofiber‐associated SCs. Mononuclear cells were filtered with a 40 μm cell strainer, followed by cell sorting using BD FACSAria Fusion Cell Sorter (BD Biosciences). BD FACSDiva (BD Biosciences, version 8.0.1) software was used to manage machine startup, data acquisition and analysis of flow cytometry data. Culture dishes were coated with poly‐D‐lysine (Sigma, P0899) and Matrigel (BD Bioscience, 356,234). FACS isolated SCs were seeded in coated culture dish and cultured in Ham's F10 medium with 10% heat‐inactivated horse serum, 5 ng/ml FGF‐Basic (AA 10–155) (Gibco, PHG0026), or cultured in differentiation medium (Ham's F10 medium with 2% horse serum and 1% penicillin/ streptomycin).

### 4.5 EdU incorporation assay

EdU incorporation assay was performed as described previously (Li et al., 2020). EdU was added to cultured MuSCs for 4 hours before fixation, followed by fixation in 4% paraformaldehyde (PFA) for 15 min and stained according to the EdU staining protocols provided by manufacturer (Thermo Fisher Scientific, C10086).

### 4.6 Single myofibers isolation and culture

Extensor digitorum longus (EDL) muscles were dissected and digested in collagenase II (800 units/ml) in DMEM medium at 37°C for 75 min. Single myofibers were released by gentle trituration with Ham's F‐10 medium with heat‐inactivated horse serum and 1% penicillin/ streptomycin, then cultured in this medium for the follow‐up experiments.

### 4.7 Genomic editing by CRISPR‐Cas9 in C2C12 cells

To delete *PAM* exon1, target‐specific guide RNAs (gRNAs) were designed using CRISPR design tool (http://crispr.mit.edu), followed by cloning into BbsI digested px330 plasmid (Addgene, 42,230). To perform genomic deletion, a pair of gRNAs containing plasmids were co‐transfected into C2C12 cells with screening plasmid pSIREN‐RetroQ (Clontech) using Lipofectamine 2000. Cells were selected with 2.5 μg of puromycin for 3 days at 48 h post‐transfection. Cells were diluted to 1 cell per well in 96 well plate. Individual colonies were PCR validated. Sequences of gRNAs and genotyping primers were listed in Table S4.

### 4.8 Plasmids

Full‐length cDNA of *Gm12603* (*PAM*) was cloned into pcDNA3.1 vector using HindIII and KpnI restriction enzymes digestion site. Primer sequences were listed in Table S4.

### 4.9 RT‐qPCR

RNAs were extracted using Trizol (Life Technologies), followed by reverse transcription using SuperScript III Reverse Transcriptase (Life Technologies). PCRs were performed with SYBR green (Life Technologies). PCR products were analyzed by LC480 II system (Roche). Primers used are listed in Table S4.

### 4.10 RNA pulldown

RNA pulldown was performed as described previously (Li et al., 2020). *PAM* DNA constructs were first linearized by single restriction enzyme digestion (NotI and XhoI for antisense and sense transcription respectively). Biotinylated transcripts were generated by these digested constructed by *in vitro* transcription using Biotin RNA labeling Mix (Roche) and MAXIscript T7/T3 *In Vitro* Transcription Kit (Ambion). Transcribed RNAs were denatured at 90°C for 2 min, then cooling on ice for 5 min, followed by addition of RNA structure buffer (Ambion) and refolding at room temperature for 20 min. Nuclear proteins from C2C12 cells were collected by resuspending cell pellet in nuclear isolation buffer (40 mM Tris–HCl pH 7.5, 1.28 M sucrose, 20 mM MgCl_2_, 4% Triton X‐100 and 1x protease inhibitor). Nuclei were collected by centrifugation at 3000 g and 4°C for 10 min. Supernatant was removed, and nuclear pellet was resuspended in 1 ml RIP buffer (25 mM Tris–HCl pH 7.4, 150 mM KCl, 0.5 mM DTT, 0.5% NP‐40, 1 mM PMSF, 1x RNase inhibitor, and 1x protease inhibitor), followed by homogenization for 10 cycles (15 s on/off) using Ika homogenizer (Ika‐Werke Instruments, Germany). Nuclear envelops and debris were removed by centrifugation at 16,200 g for 10 min. For RNA pulldown assay, 1 mg of nuclear extracts were incubated with 3 μg of refolded RNA on rotator at room temperature for 1 h. 30 μl of prewashed Dynabeads M‐280 Streptavidin were added to each reaction with incubate on rotator at room temperature for additional 1 h. Streptavidin beads were collected using a magnetic rack, and beads were washed with 1 ml RIP buffer for 5 times. Proteins were eluted by adding Western blot loading buffer and incubated at 95°C for 5 min, followed by removal of beads using magnetic rack. RNA pulldown samples were analyzed by SDS‐PAGE followed by silver staining and LC–MS/MS with Q Exactive and Easy‐nLC 1000 system (Thermo Fisher). Peptides were identified using MASCOT.

### 4.11 RNA fluorescence in situ hybridization (FISH)

RNA FISH was performed as described previously (Chen et al., 2017). Cells were fixed with 4% formaldehyde in PBS for 15 min at room temperature, followed by permeabilization with 0.5% Triton X‐100, 2 mM VRC (NEB) on ice, and two times 2x SSC wash for 10 min each. Probes were first amplified with PCR using *PAM* expression plasmid in RNA pulldown experiment. PCR products were then precipitated by ethanol, nick‐translated and labelled with Green d‐UTP (Abbott) and nick translation kit (Abbott). For each FISH experiment, 200 μg of probe and 20 μg of yeast tRNA were lyophilized and redissolved in 10 μl formamide (Ambion), followed by denaturation at 100°C for 10 min and chilled immediately on ice. Denatured probes were mixed with hybridization buffer at 1:1 ratio. 20 μl of hybridization mix was added onto fixed cells, followed by putting coverslip on it and incubated at 37°C for 16 h in a humidified chamber. Cells were then washed twice in 2x SSC, 50% formamide; thrice in 2x SSC; and once in 1x SSC for 5 min each in 42°C. Cells were mounted by coverslip with ProLong Gold Antifade Reagent with DAPI (Invitrogen). Fluorescence images were taken in Olympus microscope FV10000 and FV10‐ASW software (version 01.07.02.02, Olympus).

### 4.12 Cellular fractionation

Cellular fractionation was performed as described previously (Chen et al., 2017). C2C12 cell pellet from 1 × 10^6^ cells was lysed with lysis buffer (140 mM NaCl, 50 mM Tris–HCl pH 8.0, 1.5 mM MgCl_2_, 0.5% NP‐40, and 2 mM Vanadyl Ribonucleoside Complex) for 5 min at 4°C, followed by centrifugation at 4°C 300 g for 2 min. The supernatant after centrifugation was considered as cytoplasmic fraction and stored in −20°C for storage, while the pellet was resuspended in 175 μl resuspension buffer (500 mM NaCl, 50 mM Tris–HCl pH 8.0, 1.5 mM MgCl_2_, 0.5% NP‐40, 2 mM Vanadyl Ribonucleoside Complex) and incubate at 4°C for 5 min. Nuclear‐insoluble fraction in the resuspended pellet was removed by centrifugation at 4°C and 16,000 g for 2 min. RNA was extracted from cytoplasmic and nuclear soluble fraction by Trizol (Life Technologies).

### 4.13 Sucrose gradient

Sucrose gradient was performed as described previously (Peng et al., 2017). C2C12 cells were lysed in cell lysis buffer (50 mM Tris–HCl pH 7.6, 1 mM EDTA, 1% Triton X‐100, 10% glycerol, 1 mM DTT, 1 mM PMSF, 1x RNase inhibitor, and 1x protease inhibitor). 500 μl of whole‐cell lysate was added to 13.5 ml of 10%–30% sucrose gradient, followed by centrifugation at 38000 RPM at 4°C for 16 h. The centrifuged lysate was fractionated in 500 μl portion. To avoid cross‐contamination, only odd‐numbered fractions were obtained. Protein samples were resolved in SDS‐PAGE, followed by Western blotting. RNA samples were subjected to RT‐PCR of *PAM* and *18S*, followed by 2% agarose gel electrophoresis.

### 4.14 Chromatin immunoprecipitation using sequencing (ChIP‐seq) and ChIP‐qPCR

ChIP assays were performed as previously described (So et al., 2017). C2C12 cells were crosslinked with 1% formaldehyde at room temperature for 10 min, followed by quenching with 0.125 M glycine for 10 min. Chromatin was fragmented using S220 sonicator (Covaris), followed by incubation with 5 μg of antibodies and 50 μl Dynabeads Protein G magnetic beads (Life Technologies) at 4°C on rotator for overnight. Anti‐histone H3‐K27 acetylation (Abcam, ab4729), anti Ddx5 (Abcam, ab21696) and normal rabbit IgG (Santa Cruz Biotechnology, sc‐2027) were used in ChIP assay. Beads were washed with 1 ml RIPA buffer for 5 times, followed by decrosslinking at 65°C for 16 h and DNA extraction with phenol/chloroform. Immunoprecipitated DNA was resuspended in 50 μl of water. 200 ng of immunoprecipitated DNA was used as starting material for NEBNext® Ultra II DNA Library Preparation kit for Illumina (NEB) according to manufacturer's guideline. DNA libraries were sequenced in Illumina NextSeq 550 platform. ChIP‐qPCRs were performed with 1 μl of ChIP DNA as templated with SYBR Green Master Mix (Life Technologies), quantitative PCRs were performed on Roche LC480 system (Roche). Primers used are listed in Table S4.

### 4.15 Chromatin isolation by RNA purification using sequencing (ChIRP‐seq)

Biotin labelled probes targeting *PAM* lncRNA were designed by ChIRP Designer (LGC Biosearch Technologies) and listed in Table S4. Cells were rinsed in PBS, trypsinized, washed once with complete DMEM followed by resuspension in PBS. 10 million of ASCs were collected per ChIRP experiment for separated odd and even probe pools. Cell pellets were crosslinked with 1% Glutaraldehyde in 40 ml PBS on rotator for 10 min at room temperature, followed by quenching the crosslinking reaction with 2 ml 1.25 M Glycine for 5 min and resuspend in 1 ml chilled PBS. Cell pellets were collected at 2000RCF for 5 minutes at 4°C, followed by removing PBS, snap‐frozen with liquid nitrogen and stored at −80°C. Cell pellets were lysed and sonicated according to our standard ChIP‐seq protocol (So et al., 2017), then aliquoted into two 1 ml samples. Before ChIRP experiment, DNA were extracted for quality control with size ranging from 100‐500 bp. For ChIRP experiment, 10 μl of lysate were saved for DNA input. 1 ml of sonicated lysate was mixed with 2 ml of hybridization buffer (750 mM NaCl, 50 mM Tris–HCl pH 7, 1 mM EDTA, 1% SDS, 15% Formamide, 1x protease inhibitor and 1x RNase inhibitor). 100 pmol of odd and even ChIRP probes were added separately to the hybridization mixture and incubate at 37°C for 4 h with rotation. After the hybridization was completed, 100 μl of streptavidin magnetic C1 beads (Life Technologies, 65,001) were washed thrice with hybridization buffer and added to each ChIRP reaction for extra 30 minutes incubation at 37°C with rotation. After the hybridization completed, 1 ml of wash buffer (2X SSC, 0.5% SDS and 1x protease inhibitor) was used to wash the beads for 5 times using magnetic stand. Input control and *PAM*‐bound DNA was eluted with ChIP elution buffer for each pair of ChIRP reactions using standard elution protocol as ChIP (So et al., 2017). For ChIRP‐seq, DNA libraries were prepared as previous described in ChIP‐seq protocol (So et al., 2017). Raw reads were uniquely mapped to mm9 reference genome using Bowtie2 (Langmead & Salzberg, 2012). Peaks were called by using MACS2 (Zhang et al., 2008).

### 4.16 4C‐seq and 3C qPCR

3C experiments were performed as previously described using restriction enzyme BglII to digest fixed chromatins (Peng et al., 2017). Primers for *PAM* bait region and target regions were listed in Table S4. First round of restriction enzyme digestion in 4C‐seq was the same as 3C qPCR. 4C experiment was then continued with TatI restriction enzyme digestion, incubated overnight at 37°C and circularized using T4 DNA ligase. Gradient range of annealing temperature (55–65°C) were used to determine the optimum annealing temperature for inverse PCR. Primer sequences for inverse PCR were listed in Table S4. PCR products were subject to standard sequencing library preparation as ChIP‐seq and ChIRP‐seq. Sequencing reads with 5'end matching the forward inverse PCR primer sequence were selected and trimmed, remaining sequences containing TatI sites were mapped to mm9 assembly using Bowtie2 (Langmead & Salzberg, 2012) and the interaction regions are identified by fourSig (Williams et al., 2014).

### 4.17 RNA‐seq

Total RNAs were extracted using Trizol, followed by poly(A) selection (Ambion, 61,006) and library preparation using NEBNext Ultra II RNA Library Preparation Kit (NEB). Barcoded libraries were pooled at 10 pM and sequenced on Illumina HiSeq 1500 platform.

### 4.18 Statistical analysis

Statistical analysis of experimental data was calculated by the Student's *t*‐test, whereas * *p* < 0.05, ** *p* < 0.01, *** *p* < 0.001 and n.s. means not significant (*p* > =0.05).

## AUTHOR CONTRIBUTIONS

K.K.H.S., H.S., and H.W. designed the experiments; K.K.H.S., Y.H., and S.Z. conducted the experiments; L.H. provided support on CRISPR/cas9 experiments; Y.L. provided support on RNA pulldown experiments; X.C. and Y.Q. provided support on cellular fractionation; X.C. provided support on RNA FISH; Y.H. analyzed the sequencing data; S.Z. contributed to ex vivo muscle fiber culture; M.H.S. provided resources for molecular experiments; K.K.H.S. and H.W. wrote the paper.

## FUNDING INFORMATION

This work was supported by Collaborative Research Fund (CRF) from the Research Grants Council (RGC), University Grants Committee of the Hong Kong Special Administrative Region, China (Project Code: C6018‐19GF to H.W.); General Research Funds (GRF) from the Research Grants Council (RGC), University Grants Committee of the Hong Kong Special Administrative Region, China (Project Codes: 14106521, 14100620, 14115319, and 14106518 to H.W.; 14120420, 14116918, 14120619 and 14103522 to H.S.); Health and Medical Research Fund (HMRF) from Health Bureau of the Hong Kong Special Administrative Region, China (Project Code: 08190626 to H.W.); the research funds from Health@InnoHK program launched by Innovation Technology Commission, the Government of the Hong Kong Special Administrative Region, China; the National Natural Science Foundation of China (NSFC) to H.W. (Project codes: 82172436 and 31871304), NSFC/RGC Joint Research Scheme from the Research Grants Council, University Grants Committee of the Hong Kong Special Administrative Region to H.S. (Project code: N_CUHK 413/18); Theme‐based Research Scheme (TRS) from the Research Grants Council, University Grants Committee of the Hong Kong Special Administrative Region, China (Project code: T13‐602/21‐N); Area of Excellence Scheme (AoE) from the Research Grants Council, University Grants Committee of the Hong Kong Special Administrative Region, China (Project code: AoE/M‐402/20).

## CONFLICT OF INTEREST

The authors have declared that no conflict of interest exists.

## Supporting information


Figure S1
Figure S2
Figure S3

Table S1

Table S2

Table S3

Table S4

## Data Availability

H3K27ac ChIP‐seq, Ddx5 ChIP‐seq, *PAM* ChIRP‐seq, and *PAM* 4C‐seq using in this study have been deposited in Gene Expression Omnibus database under the accession code (GSE180073). The data that support the findings of this study are available in Gene Expression Omnibus database, reference number (GSE121589, GSE132042, GSE134529, GSE189842).
