# Supplementary figures and images for: seRNA PAM controls skeletal muscle satellite cell proliferation and aging through trans regulation of Timp2 expression synergistically with Ddx5
Source: Aging Cell. 2022 Jul 18;21(8):e13673. doi: 10.1111/acel.13673 (PMC9381903; doi:10.1111/acel.13673)

Figure S1. So K.K.H. & Huang Y. et. al.

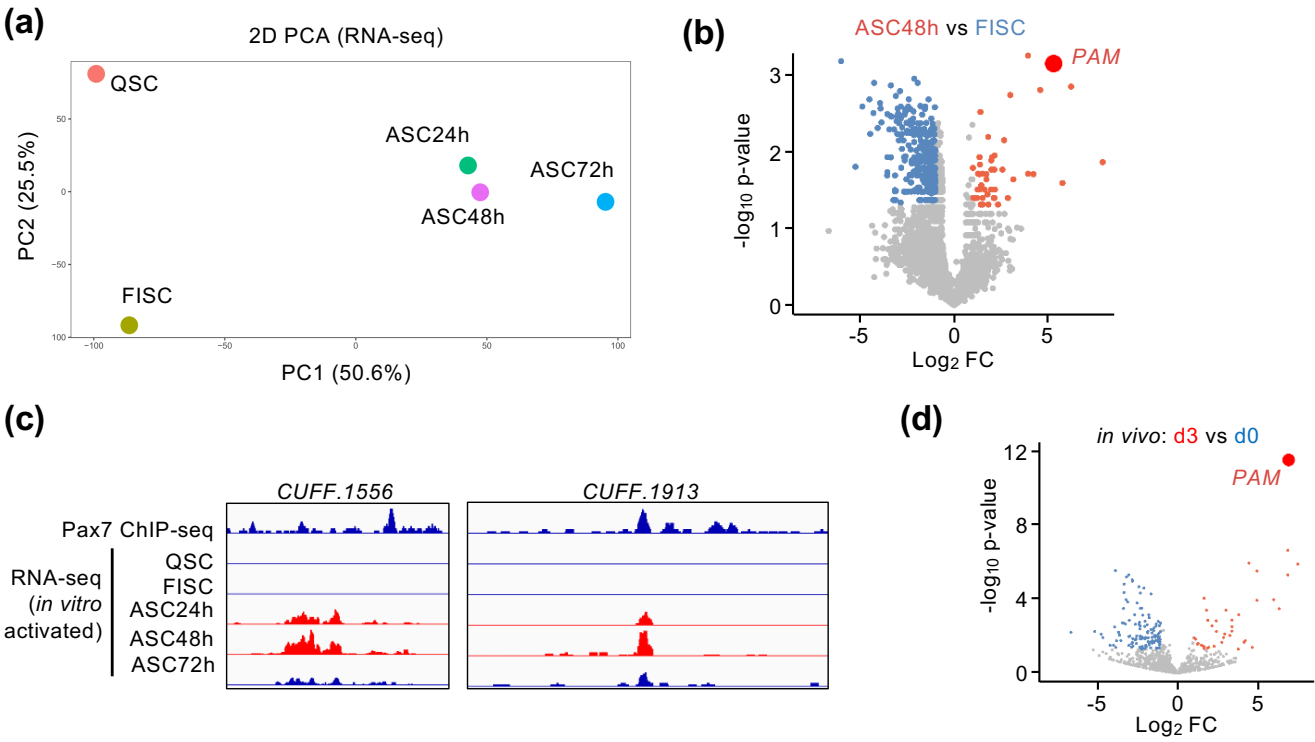

Figure S2. So K.K.H. & Huang Y. et. al.

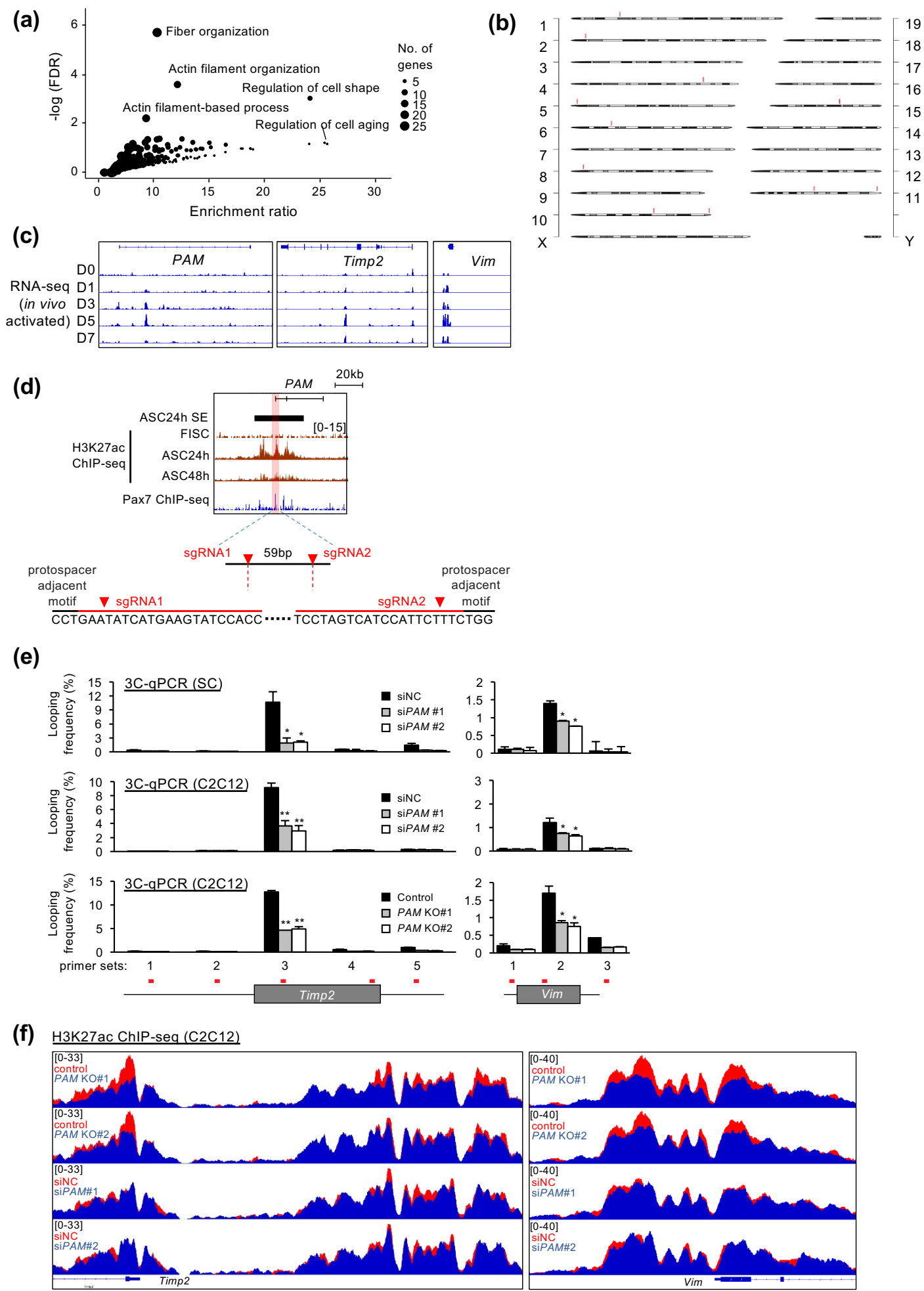

Figure S3. So K.K.H. & Huang Y. et. al.

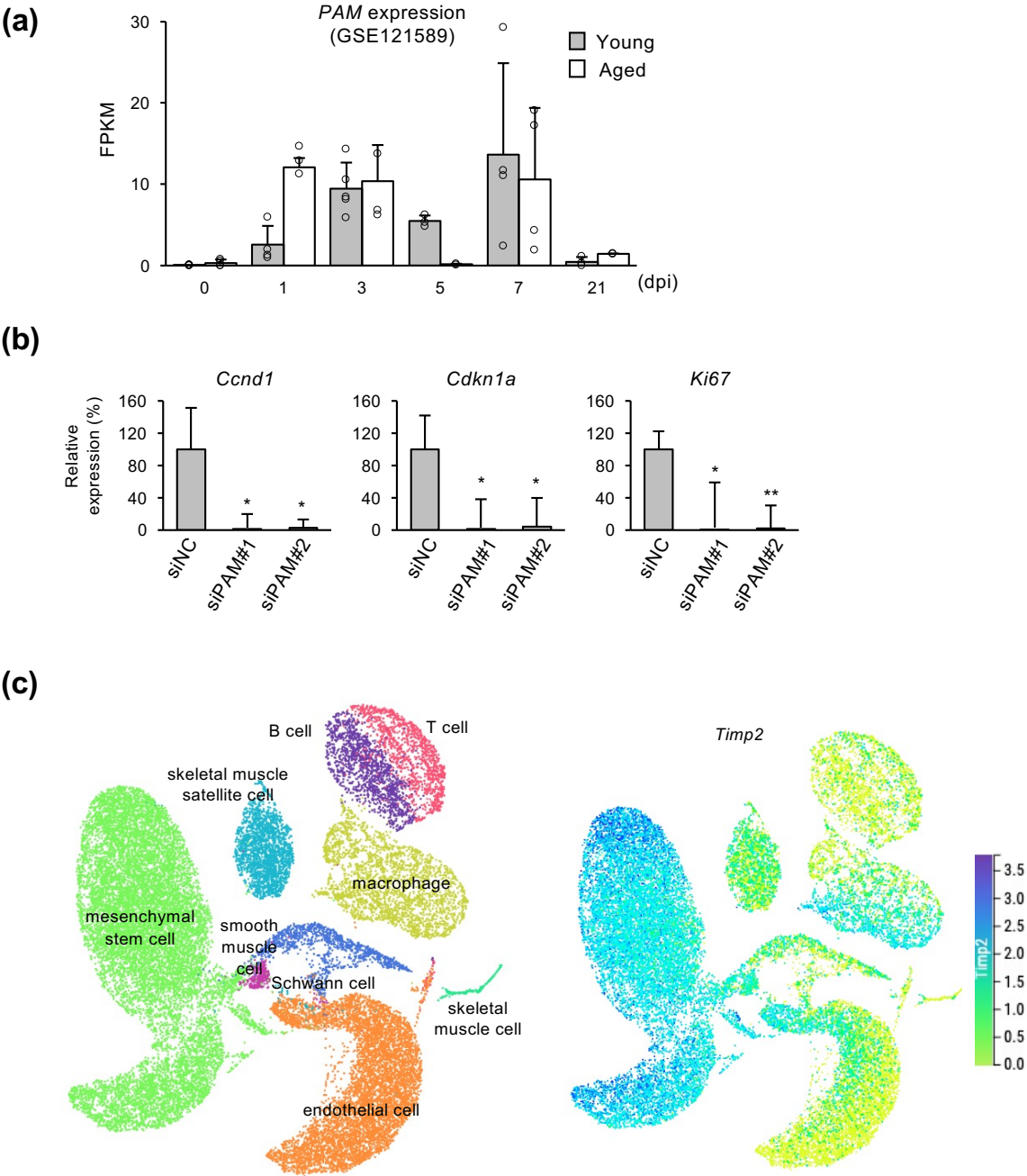

Supplement: Supplementary file 1 — Figure S1 Figure S2 Figure S3 [file ACEL-21-e13673-s004.pdf]
